# Can Antipsychotics Be the Reason for Developing Alzheimer's Disease in Schizophrenic Patients? : A Novel perspective

**DOI:** 10.1002/alz70861_108838

**Published:** 2025-12-23

**Authors:** Arwa Ayman Nagah Ahmed Shahin, Mohamed Khaled Ali Mohamed Ali Zid, Mohamed Hosni Kamel Elkellawi, Ahmed Hussein Mohamed Ismail Kira

**Affiliations:** ^1^ Cairo University Faculty of Medicine, Cairo, Cairo Egypt; ^2^ Cairo University Faculty of Medicine, Cairo, 11553 Egypt

## Abstract

**Background:**

Dopaminergic neurotransmitters have a role in the pathophysiology of both Alzheimer's disease (AD) and schizophrenia. Antipsychotics which are dopamine antagonists are widely used for the treatment of schizophrenia but have been involved in inducing cognitive impairment and death in AD patients. There is an association between schizophrenia and AD, but the cause of this is not entirely clear. This study investigates the hypothesis that schizophrenics' treatment with dopamine antagonists can lead to the development of AD in such patients.

**Method:**

We reviewed database searches through April 2025 in MEDLINE, PsycINFO, and the Cochrane Central Register of Controlled Trials; Clinical trials examining the effect of dopamine antagonists on cognitive functioning in individuals with schizophrenia and their correlation with AD were reviewed. and additional searches for ongoing trials through ClinicalTrials.gov, World Health Organization International Clinical Trials Registry Platform, and Current Controlled Trials (ISRCTN Register)

**Result:**

Patients suffering from schizophrenia carry a higher risk of developing dementia, with the long‐term treatment with antipsychotics highly probable to elevate such risk. Information indicates dopamine antagonist agents having associations with both cognitive impairment among schizophrenic patients as well as those suffering from AD and an increase in both morbidity and mortality. These findings have consequences indicating that treatment using antipsychotics could help develop AD among schizophrenics.

**Conclusion:**

Dopamine antagonists, the standard drugs for treating schizophrenia, might contribute to inducing Alzheimer's disease in patients since dopaminergic dysregulation underlies both diseases. The observation represents a call for investigating the mechanisms by which antipsychotics influence neurodegeneration more thoroughly and to come up with improved options with fewer cognitive side effects.

